
JOURNAL INFORMATION
==============================
NLM Title Abbreviation: JBRA Assist Reprod
Journal ID: JBRA Assist Reprod
NLM Journal ID: 101684552
Journal ID: jbra

JBRA Assisted Reproduction

ISSN: 1517-5693
EISSN: 1518-0557
Publisher: Brazilian Society of Assisted Reproduction

ARTICLE INFORMATION
==============================
PMCID: PMC6724381
PMID: 31436398
DOI: 10.5935/1518-0557.20190052
Article version: 1
Subjects: Poster Presentations

Abstracts of the 23rd Annual Congress of the SBRA, Curitba/PR, 2019

Publication date: 2019 Jul-Sep
Print publication date: 2019 Jul-Sep
Volume: 23
Issue: 3
First page: 301
Last page: 320
License: This is an Open Access article distributed under the terms of the Creative Commons Attribution License, which permits unrestricted use, distribution, and reproduction in any medium, provided the original work is properly cited.
License URL: https://creativecommons.org/licenses/by/4.0/

==============================


P-01. Clomiphene citrate protocol for controlled ovarian stimulation impairs endometrial maturity

Montenegro I.S. 1
Kuhl C.P. 1
Schneider R. 1
Zachia S.A. 1
Rivero R.C. 1 2
Passos E.P. 1 2
1 Hospital de Clínicas de Porto Alegre
2 Universidade Federal do Rio Grande do Sul

P-02. Two successful spontaneous pregnancies, single and twin, in uterus bicornis unicollis after deep infiltration endometriosis surgery

Tsukuda L.K. 1
Lorenzon A.R. 1
Bonetti T.C.S. 2
Serafini P.C. 1 3
Motta E.L.A. 1 2
Pereira R.M.A. 4
Domingues T.S. 1 2
1 Huntington Centro de Medicina Reprodutiva - São Paulo - SP - Brazil
2 Department of Gynecology, Paulista School of Medicine, Federal University of São Paulo, São Paulo, SP, Brazil
3 Department of Gynecology, School of Medicine, University of São Paulo, São Paulo, SP, Brazil
4 Endometriosis Center - Hospital e Maternidade Santa Joana

P-03. Darier-White disease, of the disease the healing of future generations

Borges D.D. 1 2
Ligabô Jr. M.C.N. 1 2
1 Faculdade IPEMED de Ciências Médicas e Centro In Vitro Reprodução
2 Curso de Pós-Graduação Lato Sensu em Dermatologia

P-04. Thinking ART (Assisted Reproductive Technologies): an intersectional dimension of knowledge

de Souza M.C.B. 1
Gusmão M.C.G 1
de Souza M.M. 1
Perelson S. 2
Damian A. R. 3
Bastos A.R. 4
1 Fertipraxis Centro de Reprodução Humana, RJ, Brazil
2 Universidade Federal do Rio de Janeiro -UFRJ - Brazil
3 Clinica Damian - RJ - Brazil
4 Pontifícia Universidade Católica - Rio de Janeiro - Brazil

P-05. In vitro embryonic development in differents systems: continuous, with partial or total exchange of the culture medium

Melo B.S. 1
Luna C.G. 1
Duarte V.F.F.T. 1
Donadio N.F. 1
Dzik A. 1
Cavagna Neto M. 1
1 Hospital Pérola Byington - Centro de Referência da Saúde da Mulher- SP- Brazil

P-06. Assistance in Assisted Reproduction: Who are these support professionals in Latin America? What we need?

Rito A.L.S. 1
Kimati C.T. 2
Robin F.K. 3
Repetto V. 4
Romaní Gutiérrez Y. 5
Galindo Ludeña S. 5
Cortina M.E. 6
de Souza M.C.B. 1
1 Fertipraxis Centro de Reprodução Humana - RJ, Brazil
2 Huntington Centro de Medicina Reprodutiva - SP, Brazil
3 Nilo Frantz Medicina Reprodutiva - RS, Brazil
4 Clínica Monteblanco - Chile
5 Clínica Concebir - Perú
6 Cergyr - Argentina

P-07. Safety of originator follitropin alfa (GONAL-F) for fertility treatment - Frequency of OHSS and thromboembolism in the scientific literature and the Merck KGaA safety database

Velthuis E. 1
Hubbard J. 2
Longobardi S. 3
D'Hooghe T. 3
1 Merck B.V., Global Patient Safety, Schiphol Rijk, The Netherlands
2 EMD Serono, Research & Development Institute, Billerica, U.S.A
3 Merck KGaA, Global Medical Affairs Fertility, Darmstadt, Germany

P-08. AMH as a predictor of oocyte recovery in fixed follitropin delta cycles: Preliminary data

Raupp V.A. 1
Sequeira F.F. 1
Mancebo A.C.A. 1
de Souza M.M. 1
Antunes R.A. 1
de Souza M.C.B. 1
1 Fertipraxis Centro de Reprodução Humana, RJ, Brazil

P-09. Male characteristics that impact on semen quality from percutaneous epididymal sperm aspiration (PESA) and on Assisted Reproduction outcomes

Nunes T.F. 1
Belo A. 1
Menezes N.M. 1
Lorenzon A.R. 1
Padilla C.G. 1
Motta E.L.A. 1
Alegretti J.R. 1
Wood G.J.A. 1
1 Huntington Medicina Reprodutiva, São Paulo - Brasil

P-10. Differential DNA methylation profile in men with varicocele

Santana V.P. 1
James E.R. 2
Miranda-Furtado C.L. 1 3
Souza M.F. 1
Pompeu C.P. 4
Esteves S.C. 4
Carrell D.T. 2
Aston K.I. 2
Jenkins T.G. 2
Reis R.M. 1
1 Department of Gynecology and Obstetrics, University of Sao Paulo, Ribeirao Preto, Brazil
2 Department of Surgery, University of Utah School of Medicine, Salt Lake City, USA
3 Drug Research and Development Center, Department of Surgery, Federal University of Ceara, Fortaleza, Brazil
4 ANDROFERT, Andrology and Human Reproduction Clinic, Campinas, Brazil

P-11. Use of artificial intelligence to verify gestational success

Ferreira A.S. 1
Dal Molin E.A. 1
Dos Santos J.G.C. 1
Velho J.C.M. 1
Rocha J.C. 2
1 Universidade Estadual Paulista "Júlio de Mesquita Filho" - UNESP FCL/Assis Curso de Engenharia Biotecnológica
2 Universidade Estadual Paulista "Júlio de Mesquita Filho" - UNESP. Laboratório de Matemática Aplicada - FCL/Assis - SP/ Departamento de Ciências Biológicas

Funding: FAPESP 2017 / 19323-5

P-12. Artificial intelligence applied to the prediction of human blastocysts

Fernandez E.I. 1
Miquelão M.H.C. 1
dos Santos J.G.C. 1
Chéles D.S. 1
Nogueira M.F.G. 2
1 Universidade Estadual Paulista "Júlio de Mesquita Filho", FLC - Assis; Curso de Engenharia Biotecnológica
2 Universidade Estadual Paulista "Júlio de Mesquita Filho", FLC - Assis; Departamento de Ciências Biológicas

Funding: FAPESP 2018/24252-2 2017/19323-5

P-13. Is it possible to classify human embryos, according to Gardner, through artificial intelligence?

Rocha J.C. 1
Hickman C. 2
Zaninovic N. 3
Meseguer Escrivá M. 4
Nogueira M.F.G. 1
1 Universidade Estadual Paulista "Júlio de Mesquita Filho" - UNESP. Laboratório de Matemática Aplicada - FCL/Assis - SP/ Departamento de Ciências Biológicas
2 Imperial College London, UK
3 Cornell University, New York, U.S.A.
4 Instituto Valenciano de Infertilidad, Valência, Spain

Funding: FAPESP 2017 / 19323-5

P-14. Is the presence of neoplasia associated with worse results in egg freezing?

Kira A.T.F. 1
Hentschke M.R. 1 2
Ribeiro A.T. 1
Petracco A. 1 2
Badalotti Mariangela 1 2
da Costa B.E.P. 2
1 Fertilitat - Centro de Medicina Reprodutiva
2 Pontifícia Universidade Católica do Rio Grande do Sul - PUCRS

P-15. Dual trigger is more effective than r-hCG trigger in patients with advanced maternal age and low rates of oocyte retrieval, mature oocyte and blastocyst development in previous r-hCG triggered ICSI cycles

Maldonado L.G.L. 1
Setti A.S. 1 2
Braga D.P.A.F. 1 2
Iaconelli Jr. A. 1 2
Borges Jr. E. 1 2
1 Fertility Medical Group - São Paulo -Brazil
2 Instituto Sapientiae - Centro de Estudos e Pesquisa em Reprodução Assistida - São Paulo -Brazil

P-16. Day four embryo transfer: A viable alternative

Martello C.L. 1
Roos Kulmann M.I. 1
Dutra C.G. 1
Pagnoncelli N. 1
Ferreira M. 1
Frantz N. 1
1 Nilo Frantz Medicina Reprodutiva, Porto Alegre, RS, Brazil

P-17. Mathematical variables originated from embryonic morphology and kinetics can predict the birth of living?

Chéles D.S. 1
Dal Molin E.A. 1
Hickman C. 2
Nogueira M.F.G. 1
1 Universidade Estadual Paulista, UNESP FCL-Assis, Departamento de Ciências Biológicas
2 Imperial College London, UK

Funding: FAPESP 2018/19053-0 2017/19323-5

P-18. Clinical outcomes of the shared egg donation program

Vilela F.V. 1
Coelho G.M. 1
Almeida A.S. 1
Rocha I.A. 1
Ramos T.P. 1
1 Valencian Institute of Infertility - IVI Salvador, Bahia, Brazil

P-19. Through the morfocinetic data in conjunction with the technique of artificial neural networks, is it possible to determine live births?

Cecilio M.H.M. 1
Fernandez E.I. 1
dos Santos J.G.C. 1
Hickman C. 2
Rocha J.C. 3
1 Universidade Estadual Paulista "Júlio de Mesquita Filho" (UNESP) - Faculdade de Ciências e Letras (FCL) / Assis. Curso de Engenharia Biotecnológica
2 Imperial College London, UK
3 Universidade Estadual Paulista "Júlio de Mesquita Filho" (UNESP) - Faculdade de Ciências e Letras (FCL) / Assis. Departamento de Ciências Biológicas

Funding: FAPESP 2018/19371-2 2017/19323-5

P-20. Reduced expression of HDAC2 and EHMT2 genes in men with varicocele

Souza M.F. 1
Santana V.P. 1
Biagi Jr. C.A.O. 1
Esteves S.C. 2
Miranda-Furtado C.L. 1 2
Reis R.M. 1
1 Department of Gynecology and Obstetrics, University of Sao Paulo, Ribeirao Preto, Brazil
2 Drug Research and Development Center, Department of Surgery, Federal University of Ceara, Fortaleza, Brazil

P-21. Analysis of paternal factors and embryo aneuploidy rate

Nunes T.F. 1
Belo A. 1
Moreti N. 1
Domingues T.S. 1
Lorenzon A.R. 1
Alegretti J.R. 1
Motta E.L.A. 1
Wood G.J.A. 1
1 Huntington Medicina Reprodutiva, São Paulo - Brazil

P-22. The relationship between endocrine disruptors and female infertility

Rocha A.M.M.S.V. 1
de França B.K.D. 1
de Morais G.B 1
de Araújo A.G.D. 2
1 Faculty of Medicine. University Center of João Pessoa-UNIPÊ.
2 Faculty of Medicine Nova Esperança.

P-23. Microbiological monitoring in assisted reproductive clinics

Borges E.D. 1
Berteli T.S. 1
Reis T.F. 2
da Silva A.S. 1
Vireque A.A. 3
1 Faculdade de Medicina de Ribeirão Preto da Universidade de São Paulo (FMRP-USP)
2 MicroControl LTDA - Supera Parque/USP Ribeirão Preto
3 Invitra Tecnologia da Reprodução Assistida LTDA

P-24. Fertility in patients with gynecological neoplasias: How to preserve it

Rocha A.M.M.S.V. 1
de Morais G.B. 1
de França B.K.D. 1
de Araújo A.G.D. 2
1 Faculty of Medicine. University Center of João Pessoa-UNIPÊ.
2 Faculty of Medicine Nova Esperança.

P-25. Epidemiological profile of men looking for clinical information in a Center of Reproductive Medicine in Rio Grande do Sul

Donatti L.M. 1
Oliveira N.P. 1
Dal Magro B.M. 1
Frantz G.N. 1
Frantz N. 1
1 Nilo Frantz Medicina Reprodutiva, Porto Alegre, RS, Brazil

P-26. Post-mortem Fertilization - How is the judicialization of Brazil in relation to the first world countries

Rocha A.M.M.S.V. 1
de Araújo A.G.D. 2
de França B.K.D. 1
de Morais G.B. 1
1 Faculty of Medicine. University Center of João Pessoa-UNIPÊ.
2 Faculty of Medicine Nova Esperança.

P-27. Chromosomal abnormalities analysed by NGS platform + STRS in products of conception of 1st and 2nd trimester pregnancy losses

Ali T. 1
Motta P. 1
Uehara M. 1
Cervera A. 2
Al-Asmar Piñar N. 2
Riboldi M. 1
1 Igenomix Brazil - Sao Paulo - Brazil
2 Igenomix SL - Valencia - Spain

P-28. Transfer of one euploid blastocyst embryo versus two non analysed blastocyst embryos: Preganancy and abortion rate influence

Tagliani-Ribeiro A. 1
Flach S.W. 1
Wingert F. 1
Azambuja R. 1
Petracco A. 1 2
Badalotti M. 1 2
1 Fertilitat - Centro de Medicina Reprodutiva - Porto Alegre
2 Departamento de Ginecologia e Obstetrícia da Faculdade de Medicina da PUCRS - Porto Alegre

P-29. Comparative analysis of risk factors to poor ovarian response to stimulation and clinical outcomes of this subgroup in IVF cycles

Amaral M.E.B. 1
Monteleone P.A.A. 1
Ejzenberg D. 1
Serafini P. 1
Soares Jr. J.M. 1
Baracat E.C. 1
1 Disciplina de Ginecologia, Departamento de Obstetrícia e Ginecologia, Hospital das Clínicas, Faculdade de Medicina, Universidade de São Paulo, São Paulo, Brazil.

P-30. Oxidative metabolism and embryonic development of oocytes treated with lipid molecules and antioxidants during vitrification: Preclinical study

Viana I.G.R. 1
Vireque A.A. 2
Navarro P.A.A.S. 1
1 Faculdade de Medicina de Ribeirão Preto da Universidade de São Paulo (FMRP-USP)
2 Invitra Tecnologia da Reprodução Assistida LTDA - Supera Parque/USP

P-31. FIV/ICSI - Initial results of oocytes accumulation in our clinic

Borges G.C. 1
Florencio R.S. 1
de Oliveira V.A. 1
Camarço M.N.C.R. 1
Finotti M.C.C.F. 1
de Castro E.C. 1
1 Humana Medicina Reprodutiva- Goiania- GO- Brazil

P-32. Empirical use of Assisted Hatching does not improve the overall IVF clinical outcomes.

Damasceno T. 1
Nazareth L. 1
Vaz B. 1
Pontes A.C. 1
Prates N. 1
Roque M. 1
Valle M. 1
1 ORIGEN, Rio de Janeiro

P-33. Influence of blastomere asymmetry on blastocysts quality and pregnancy rate of good responders.

de Souza L.K. 1
da Cunha Filho J.S.L. 1
1 Center of Human Reproduction Insemine, Porto Alegre/RS, Brazil.

P-34. Frequency of findings in the carrier screening in Brazilian individuals

Motta P. 1
Ali T. 1
Mir P. 2
Martín J. 2
Riboldi M. 1
1 Igenomix Brazil - São Paulo - Brazil
2 Igenomix SL - Valencia - Spain

P-35. Double versus single lumen needle: Still worth this question?

de Souza M.M. 1
Mancebo A.C.A. 1
Barbeitas A.L.O. 1
Antunes R.A. 1
Areas P.C.F. 1
de Souza M.C.B. 1
1 Fertipraxis Centro de Reprodução Humana, RJ, Brazil

P-36. Single embryo transfer: Comparison between single x double transfer of blastocysts

Araújo Filho E. 1
de Araújo L.P. 1
Fácio C.L. 1
Machado-Paula L.A. 1
Previato L.F. 1
1 Centro de Reprodução Humana de São José do Preto (CRH Rio Preto), São José do Rio Preto, SP, Brasil

P-37 Relation between the morphology of the spermatozoa head and the flagellum edema class after the hyposmotic test (HOST)

Araujo D.M. 1
da Silva A.S. 1
Pereira D.M.M. 1
Zanini V. 1
Giusti A.M. 1
Amara V.L.L. 1
1 Universidade do Vale do Itajaí (UNIVALI)

P-38. Motivation of men to be semen donors

dos Santos J.R. 1
Feitosa T.V. 1
de Oliveira B.B.P. 1
Yoshida I.H. 1
Barbosa C.P. 1
1 Instituto Ideia Fértil de Saúde Reprodutiva

P-39. Follicular output rate e motilidade espermática pós-processamento estão relacionados com a ocorrência de falha total de fertilização

Mattia M.M.C. 1
de Souza L.K. 1
Bessow C.K. 1
Donato R.C. 1
Cunha-Filho J.S. 1
1 Center of Human Reproduction Insemine, Porto Alegre/RS, Brazil.

P-40. Impact the in vitro culture of isolated bovine preantral follicles on the recovered oocytes quality

Batista LA. 1
Silva L.C.C. 1
Higa T.T.
Molina C.P.P. 1
Lima C.A.C. 1
Alberici L.C. 1
Rosa-E-Silva A.C.J.S. 1
1 Departamento de Ginecologia e Obstetrícia - Faculdade de Medicina- Universidade de São Paulo -Ribeirão Preto- Brazil

P-41. Correlation of TSH levels with ovarian reserve and reproductive outcomes in women undergoing in vitro fertilization

Bessow C.K. 1
Donato R.C. 1
T.O. 1
de Souza L.K. 1
da Cunha Filho J.S.L. 1 2
1 Centro de Reprodução Humana Insemine
2 Universidade Federal do Rio Grande do Sul

P-42. Can the use of perfume influence in vitro embryonic development?

Araujo D.M. 1
Salvador R.A. 1
Giusti A.M. 1
Til D. 1
Senn A.P. 1
Amara V.L.L. 1
1 Universidade do Vale do Itajaí (UNIVALI).

P-43. ERA results in women with recurrent implantation failure in Brazil

Ali T. 1
Uehara M. 1
Alonso M.R. 2
Riboldi M. 1
1 Igenomix Brazil - Sao Paulo - Brazil
2 Igenomix SL - Valencia - Spain

P-44. Embryo symmetry in women with and without endometriosis undergoing IVF

Bessow C.K. 1
Donato R.C. 1
de Souza T.O. 1
Mattia M.M.C. 1
de Souza L.K. 1
da Cunha Filho J.S.L. 1 2
1 Centro de Reprodução Humana Insemine
2 Universidade Federal do Rio Grande do Sul

P-45. Tubal perforation due to hysterosalpingography: a case report

Moro A.P. 1
Repukna B.G. 1
Piotto K.L. 1
Fiore G.A.D. 1
Righi M. 1
1 Universidade Estadual do Oeste do Paraná - Campus Francisco Beltrão - PR.

P-46. In vitro fertilization with egg donation: preliminary results of a retrospective cohort of 15 years

Colombo T. 1
Kira A. 1
Hentschke M. 2
Badalotti M. 1
Farinati D. 1
Wendland E. 3
1 Fertilitat Centro de Medicina Reprodutiva
2 Pontifícia Universidade Católica do Rio Grande do Sul - PUCRS
3 Universidade Federal de Ciências da Saúde de Porto Alegre - UFCSPA

P-47. In vitro embryonic development in different systems: continuous, with partial or total exchange of the culture medium

Salvador R.A. 1
Araujo D.M. 1
Nunes J.L. 1
Senn A.P. 2
Til D. 1
Amaral V.L.L. 1
1 Universidade do Vale do Itajaí (UNIVALI).
2 Fertas Brasil

P-48. Azoospermia in Fabry's disease and Assisted Reproduction: Case report

Moreno V.C.F. 1
Figueira R.C.S. 1
Verza Jr. S. 1
Esteves S.C. 1
1 ANDROFERT - Centro de Referência para Reprodução Masculina - Campinas - SP

P-49. Duostim cycles potentially boost reproductive outcomes in poor prognosis patients

Cecchino G.N. 1 2 3 4
Roque M. 4 5
Cerrillo M. 3
da Rosa Filho R. 4
Chiamba F.S. 4
García-Velasco J.A. 2 3
1 Federal University of São Paulo, Department of Gynecology, São Paulo-SP, Brazil;
2 Rey Juan Carlos University, Department of Gynecology and Obstetrics, Madrid, Spain
3 IVIRMA Global Madrid, Madrid, Spain.
4 Mater Prime, São Paulo-SP, Brazil.
5 Origen - Center for Reproductive Medicine, Rio de Janeiro-RJ, Brazil.

P-50. Effect of overweight and obesity in clinical outcomes following in vitro fertilization procedures

Hentschke M.R. 1
Dornelles V.C. 1
Antunes V.B. 1
Cunegatto B. 2
Colombo T. 2
Badalotti M. 2
1 Faculdade de Medicina da PUC-RS (Pontifícia Universidade Católica do Rio Grande do Sul).
2 Fertilitat Centro de Medicina Reprodutiva.

P-51. DNA mitocondrial e seu valor preditivo na implantação de blastocistos euplóides predictive value of mitochondrial dna ratio on euploid blastocyst implantation potential

Moreno V.C.F. 1
Figueira R.C.S. 1
Verza Jr. S. 1
Esteves S.C. 1
1 ANDROFERT - Centro de Referência para Reprodução Masculina - Campinas - SP

P-52. Progestin-primed ovarian stimulation (PPOS) during follicular versus luteal phase: A comparison of 100 IVF/ICSI cycles

Lemos Nakano M.S. 1
Ramos F.O. 1
Oliveira V.H.B. 1
Duarte Filho O.B. 1
Tomioka R.B. 1
Yamakami L.Y.S. 1
1 Vida Bem Vinda Clinic, Department of Reproductive Medicine, São Paulo, Brazil.

P-53. Influence of BMI and age in IVF patients

Ribeiro K.H. 1
Cerqueira M.H.R. 1
Silva G.C. 1
Perez V.N. 1
Esteves A.C.C. 1
1 Clinifert - Centro de reprodução Humana- Florianópolis- Brazil.

P-54. Influence of gonadotrophin dose in embryonic ploidy

Carneiro M. 1
Kozlowski I. 1
Campos B.C.V. 1
da Rosa V.B. 1
Schuffner A. 1
1 Clinica Conceber - Centro de Medicina Reprodutiva.

P-55. AMH And number of oocytes: prognosis and conduct suitable for treatment and in vitro fertilization

Kozlowski I. 1
Carneiro M. 1
Campos B.C.V. 1
Rosa V.B. 1
Schuffner A. 1
1 Conceber Centro de Medicina Reprodutiva - Curitiba, PR

P-56. Efficiency of obtaining blastocysts in different age groups and pregnancy rate

Rosa V.B. 1
Campos B.C.V. 1
Schuffner A. 1
1 Conceber Centro de Medicina Reprodutiva - Curitiba, PR

P-57. Culture from zygotto to blastocyst through the use of bicabornate buffer system GV BLAST™

Morais L.P. 1
Rodrigues N.L.S. 1
Rogério L.S.R. 1
de Souza L.R. 1
1 Clínica La Vitta - Centro de Reprodução Humana do Amazonas, Manaus-AM.

P-58. Development and validation of a protocol for analysis of sperm DNA fragmentation

Giusti A.M. 1
Siewert A. 1
Araujo D.M. 1
Rosas I.M. 1
Salvador R.A. 1
Amaral V.L.L. 1
1 Universidade do Vale do Itajaí (UNIVALI).

Subjects: Articles

P-59. Quantitative analysis of phospholipids and triacylglycerol lipids by multiple reaction monitoring profiling (MRM-PROFILING) in oocyte and embryo samples

Xie Z. 1
Ferreira C.R. 1
Vireque A.A. 2
Sobreira T.J.P. 1
Cooks R.G. 1
1 Purdue University, West Lafayette, IN
2 Invitra, Assisted Reproductive Technologies Ltd., Ribeirão Preto, Brazil

Support: FAPESP (Processes 2017 / 19323-5) and CNPQ bolsa PIBIC / UNESP.

Support: FAPESP (Processes 2018/24252-2 and 2017/19323-5).

Support: FAPESP (Process 2017 / 19323-5).

Support: FAPESP (Processes 2018/19053-0 and 2017/19323-5).

Support: FAPESP (Processes 2018/19371-2 and 2017/19323-5).

